# Analysis of Chinese consumers’ willingness and behavioral change to purchase Green agri-food product online

**DOI:** 10.1371/journal.pone.0265887

**Published:** 2022-04-06

**Authors:** Longbo Ma, Ziyu Li, Dan Zheng

**Affiliations:** 1 School of Economics and Management, Qingdao Agricultural University, Qingdao, Shandong, China; 2 Bathers Institute of Future Agricultural Technology, Qingdao Agricultural University, Qingdao, Shandong, China; Murdoch University, AUSTRALIA

## Abstract

Internet coupled with agricultural development in full swing, green agri-food product e-commerce has become a hot topic in the development of e-commerce. However, due to the seasonality, territoriality, weak quality, difference, and dispersion of agri-food products, e-commerce of green agri-food products are facing difficulties. Based on the gap between consumers’ willingness and behavior to buy green agri-food products online, this paper quantitatively studied the factors that affect consumers’ behavior deviation to buy green agri-food products online. The method of constructing the binary Logistic regression model was adopted, and analysis tools such as SPSS and Excel were used for analysis, to study the influence of various factors on consumers’ purchase willingness and behavior. The research conclusion shows that: consumers’ gender, age, understanding of online agri-food products, and monthly disposable income have a positive impact on the consistency of consumers’ behavior and willingness, while the price of green agri-food products and online purchasing frequency have a negative impact on consumers’ willingness and behavior of online purchasing. The results are discussed in the context of existing studies. Suggestions and research prospects are presented at the end of the article.

## Introduction

With the coming of the “Internet Plus” era, online purchasing plays an increasingly important role in economic development around the world. The enhancement of consumers’ attention to food safety promotes the increasing proportion of green agri-food products in online purchasing. In particular, the e-commerce model of green agri-food products is growing quickly in some regions of China with the improvement of rural infrastructure, information construction, and relevant policies. Many platforms have emerged to accelerate the development of the e-commerce model of green agri-food products in China. The e-commerce model of green agri-food products is contributed to the transformation of modern agriculture, the improvement of farmers’ livelihood, and the realization of rural revitalization.

The Chinese government has issued several national policies to support the e-commerce model development of green agri-food products. However, there are many problems still restrict the development of the e-commerce model of green agri-food products, including consumers’ trust, quality assurance, and storage cost. In addition, consumers have a higher perceived risk for green agri-food products during online purchasing due to their lack of physical perception of product quality, which has negative impacts on the development of e-commerce models of green agri-food products. Hence, it is urgent to analyze the deviation between consumers’ willingness and behavior and its influencing factors, to provide the basis for promoting the development of the e-commerce model of green agri-food products.

Consumers’ food purchase decision is multifaceted and complex, which is not only influenced by product and process characteristics, but also by the present decision-making circumstances. Among the nine food purchase criteria (e.g. country-of-origin (CoO) labeling, production methods, chemical residue testing (CRT) information, price, shopping location, visual appearance, sense of touch, package size, and a recommendation given by a significant one), CRT information is the most important criteria in consumers’ purchase decision, followed by CoO, production methods and hedonic characteristics [1]. The study of consumers’ willingness to pay for organic, clean label, and processed with a new food technology highlight the importance of technological innovations and the need of presenting consumers with complete information on the new food technologies to mitigate potential neophobias due to unfamiliarity [2]. As to organic food, attitude and health consciousness are found to be better predictors of organic food purchase intention. The awareness of consumers moderates positively in the intention to purchase organic food [3]. Besides attitude, subjective and personal norms and (perceived) behavioral control influence the consumption of organic food [4].

It was found that, in terms of influencing factors affecting consumers’ willingness to purchase green agri-food products, consumers’ perceptions of the health attributes of green agri-food products and the environmental role of green agri-food products, and the marketing efforts of companies are the significant influencing factors [5]. There are differences in the factors influencing consumers’ purchase of green agri-food products in different levels of cities, but personal characteristics factors are the deep-seated factors influencing consumers’ willingness to purchase green agri-food products [6]. In terms of influential factors affecting consumers’ behavior in purchasing green agri-food products, the cognitive attitude factor has the greatest impact on consumers’ purchasing behavior [7], and the willingness to consume, ease of purchase, and government regulation of green agri-food products have significant effects on green agri-food products consumption behavior [5]. In addition to traditional economic variables such as income and price, consumer attention, trust, and willingness to pay will also have an important impact on purchasing behavior; information asymmetry, lack of consumer confidence and high premium levels are the main reasons for refusal to buy [8]. In general, the factors affecting consumers’ willingness or behavior to purchase agri-food products online can be generally divided into the following five aspects: individual characteristics of consumers, economic characteristics, cognition of online agri-food products, factors of demand, and motivation, and factors of purchasing atmosphere [9].

In recent years, the rise of online shopping and the development of the logistics and transportation industry has brought a new development direction to the sales and production of agricultural products, and Zhan believes that the online sales of agricultural products have considerable multiplier effects in terms of improving market competitiveness and product brand awareness [10]. Shang believes that China’s GI agricultural products started late and there are certain problems in the development process, such as the incomplete legal system regarding the online sales of agricultural products [11]. In this regard, experts have also done relevant studies, including the current development of the online shopping industry for agricultural products [12], online business model [13], cold chain logistics and transportation system [14], and aspects of consumers’ preferences for online shopping of agricultural products. Regarding the factors that influence consumers’ online shopping intention and behavior, they are mainly divided into the following aspects: personal characteristics of consumer groups, attitudes toward online shopping, characteristics of sales websites, characteristics of agricultural products, and online shopping preferences [15].

Studies have found that consumers will have inconsistencies between their intention and behavior when making purchases. Jin pointed out that even though consumers have high consumption intentions, they do not translate them into actual consumption behavior to a large extent, which means that there are significant differences in consumption intentions and behavior. As for the factors that influence the difference in willingness behavior, the main ones are individual characteristics, product perception, supply channel income level, product price, quality certification, and social environment [16].

Overall, previous research more focused on the influencing factors of consumers’ online purchasing of agri-food products, while the influencing factors of the transformation of purchasing willingness to the purchasing behavior of agri-food products remain unclear. Under the background of the new retail era, this paper studies the transformation of consumption intention and behavior of green agri-food products based on the framework of perceived benefit and perceived risk. Specifically, this paper aims to: a) study the deviation between consumers’ online purchasing intention and actual purchase behavior of agri-food products, and predict the purchase behavior of the consumers from the perspective of behavioral economics plan; b) quantitatively analyze the deviation in consumer intention and behavior, and explore its correlation with basic characteristics, and c) study the factors influencing consumer agri-food products online purchasing intention and behavior deviation, and some suggestions countermeasures.

## Materials and methods

### Theoretical model

**Fig 1:**
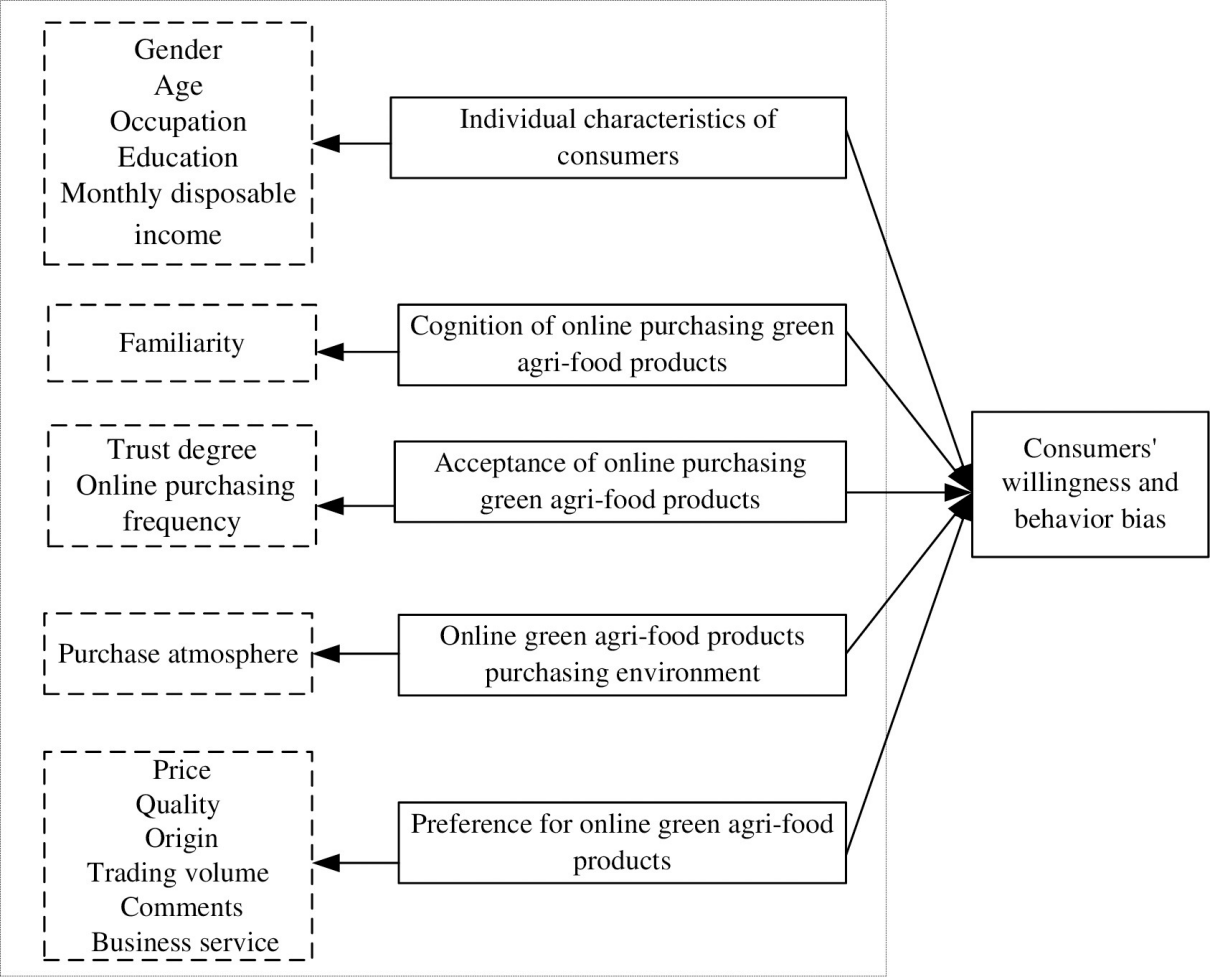
The basic framework of consumers’ willingness and behavior to purchase green agri-food products.

According to the theory of planned behavior, individual cognition is mainly represented by behavioral attitude, subjective norm, and perceived behavior. Previous research showed that individual characteristics of the consumers significantly affect their willingness and behavior on purchasing green agri-food products, which include cognition, agri-food product acceptance, agri-food product atmosphere and the preference for online purchasing, etc. Based on the above analysis, this study constructed the basic framework of consumers’ willingness and behavior change to purchase green agri-food products in the new retail era (Fig 1).

#### Individual characteristics of consumers

Individual characteristics include the gender, age, occupation, education, and monthly disposable income of the consumers [17, 18]. The studies found that the consistency of behavior and willingness of these consumers was significantly higher than that of other categories, including the ages between 18 and 30, the female consumers, the education with an undergraduate degree, the students, and the monthly disposable income between 1000 and 2000 [19].

#### Cognition of online purchasing green agri-food products

The consumers’ cognition degree of online purchasing green agri-food products could be divided into 5 levels, including completely unknown, not very familiar, general, relatively well familiar, and very well understood [19]. The consistency of consumer willingness and behavior would be increased with the degree of details on the frequency of online purchasing green agri-food products.

#### Acceptance of online purchasing green agri-food products

The consumers’ acceptance of online purchasing green agri-food products could be represented by trust degree and frequency [19, 20]. Whether consumers trust the quality of real estate green agri-food products will influence their consumption behavior [21]. According to consumers’ consumption habits, the purchase possibility of the consumers would increase with the degree of acceptance and trust in the online green agri-food products. In terms of consumer online purchasing, the deviation of behavior and willingness of the consumers would be decreased with the degree of acceptance and trust on the online purchasing.

#### Online green agri-food products purchase atmosphere

Li pointed out that in an online shopping environment, reference groups (expressed by the number of people who have purchased online) significantly and positively affect consumers’ perceived value and willingness to purchase online [22]. In contrast, a reference group is a person or group that has direct contact with an individual, or an individual or group that does not have direct contact but will have an impact on the individual [23]. Through investigation, the frequency of people around buying products online can be adopted to understand the consumer purchasing atmosphere around. The points of five degrees include rarely, less, generally, more, and a lot. It is generally believed that consumers have the online purchasing behavior that will also influence others surrounded.

#### Preference for online green agri-food products

Preferences can influence a buyer’s purchase decision and also have an impact on the purchase decision [24]. In this paper, based on previous research results and the current sales status of agri-food products on the online platform, six variables, product price, quality and place of origin, product trading volume, online evaluation, and seller’s service attitude, are selected as preference factors [25].

### Model construction

There are many methods that can be used to study the above problems, such as multiple regression model, structural equation model, etc. In this paper, "consumers’ willingness to purchase agricultural products online and behavioral deviation" is used as the dependent variable to investigate the factors influencing consumers’ willingness to purchase agricultural products online and behavioral deviation, so this paper regards the positive consistency and negative consistency of willingness and behavior as consistent, and the deviation of willingness and behavior as inconsistent, so the dependent variable in this study is a dichotomous variable, and it is suitable to use the binary logistic model for analysis, the regression analysis model is,

$$prob(y=1)=P=\frac{\beta_{0}+\sum_{i=1}^{17}\beta_{i}x_{i}}{1+\exp(\beta_{0}+\sum_{i=1}^{17}\beta_{i}x_{i})}=\frac{1}{1+\exp[-(\beta_{0}+\sum_{i=1}^{17}\beta_{i}x_{i})]}$$ (1)

Where *y* was the deviation of consumers’ willingness and behavior to purchase green agri-food products online. *P* was the probability of consumers’ willingness and behavior to purchase agri-food products online; *x*_*i*_ was the explanatory variable; *β*_*i*_ was the effective coefficient of explanatory variable; *β*_0_ was the constant term.

Transform Eq (1):

$$\frac{P}{1‐P}=\exp\;(\beta_{0}+\sum_{i=1}^{17}\beta_{i}x_{i})$$ (2)

The logarithm of both sides of Eq (2) is taken to obtain the final regression model as follows:

$$y=Ln(\frac{P}{1‐P})=\beta_{0}+\sum_{i=1}^{17}{}_{i}\beta X_{i}$$ (3)

### Data source

The study and the associated data sourcing approach are approved by the Research Administration Department, Qingdao Agriculture University. The survey was conducted between December 2019 and May 2020 and was based on an online questionnaire, supplemented by a portion of fieldwork. The research data originated from the survey in six cities of Shandong Province in China. The questionnaire included consumers’ characteristics, online purchasing frequency of green agri-food products and its related perception. Firstly, six cities in different locations were selected using the multi-stage sampling method, including Weihai, Yantai, Qingdao, and Weifang in the eastern region, Jinan, and Jining in the central and western regions. Meanwhile, each city chose 4 townships, and each township selected 2 villages for the survey. Secondly, the sample farmers were selected using the method of equidistant sampling (k = 10) based on the village map. Equidistant sampling was carried out to supplement the number of samples if the farmers were away from home for a long time. Finally, the peasant household survey was conducted based on the questionnaire. A total of 1150 questionnaires were in the survey, and the valid questionnaire was 1090 with an effective rate of 94.78% (Table 1).

**Table 1:** Regional distribution of samples.

Region	Sample size (family)	Number of the valid sample(family)	Effective sample rate(%)
Weihai	210	199	94. 76
Yantai	202	191	94. 55
Qingdao	400	379	94. 75
Weifang	120	114	95. 00
Jinan	134	127	94. 78
Jining	84	80	95. 24
Total	1150	1090	94. 78

### Variable characteristic

The descriptive statistics of each variable were shown in Table 2.

**Table 2:** The descriptive statistics of each variable.

	The variable name	Interpretation of variables and descriptive analysis	Average	The standard deviation	The minimum value	The maximum
The dependent variable	Willingness and behavior bias	0 = disobey, 1 = positive consistency, 1 = negative consistency	0.66	0.48	0	1
Personal Characteristic	Gender	0 = woman; 1 = man	1.5	0.5	0	1
	Occupation	1 = students, 2 = farming, 3 = migrant workers, 4 = freelance work, 5 = other	2.51	1.28	1	5
	Age	1 = under 20 years old, 2 = 20 to 35 years old, 3 = 35 to 40 years old, 4 = 40 to 45 years old, 5 = 45 years of age or older	2.86	1.03	1	5
	Education	1 = senior high school and below, 2 = junior college or undergraduate, 3 = postgraduate and above	2	2	1	3
	Monthly disposable income	1 = 2000 RMB and under, 2 = 2000~4000, 3 = 4000~6000, 4 = more than 6000 RMB	2.27	2.27	1	4
Cognitive degree	Familiarity	1 = can’t understand; 2 = General understanding; 3 = Fully understand,	1.85	2.27	1	3
Acceptance of online purchasing	Online purchasing frequency	1 = never, 2 = once a month or less, 3 = twice a month, 4 = three times or more per month	2.15	2.15	1	4
	The degree oftrust	1 = In complete disbelief, 2 = Less convinced, 3 = general, 4 = A little believe, 5 = Very believe	2.94	2.94	1	5

Purchas environment	Purchase atmosphere	1 = rarely, 2 = lesser, 3 = ordinary, 4 = there are many, 5 = a great money	3. 16	3. 16	1	5
Preference for online agri-food products	Price	1 = pay no attention to, 2 = take less seriously, 3 = ordinary, 4 = more attention to, 5 = Attaches great importance to	3.03	3.03	1	5
	Quality	1 = pay no attention to, 2 = take less seriously, 3 = ordinary, 4 = more attention to, 5 = Attaches great importance to	3.5	3.5	1	5
	Origin	1 = pay no attention to, 2 = take less seriously, 3 = ordinary, 4 = more attention to, 5 = Attaches great importance to	2.83	2.83	1	5
	Comments	1 = pay no attention to, 2 = take less seriously, 3 = ordinary, 4 = more attention to, 5 = Attaches great importance to	3.43	3.43	1	5
	Trading volume	1 = pay no attention to, 2 = take less seriously, 3 = ordinary, 4 = more attention to, 5 = Attaches great importance to	2.99	2.99	1	5
	Business services	1 = pay no attention to, 2 = take less seriously, 3 = ordinary, 4 = more attention to, 5 = Attaches great importance to	3.12	3.12	1	5

Data source: According to the statistical collation of the survey questionnaire

## Results

This study conducted the correlation analysis to explore the relationship between different variables and willingness and behavior bias. Results showed that only sex, occupation, age, education, and monthly disposable income of consumers had the higher correlations relationship with their willingness and behavior bias. Hence, this study only analyzed the influencing degrees of the above 5 variables to willingness and behavior bias using the Logistic regression model.

### Cross analysis

There were 450 consumers whose purchasing willingness and behavior were consistent, accounting for 41.28% of the surveyed samples. 270consumers whose purchasing willingness and behavior were negatively consistent, accounting for 24.77% of the surveyed samples. Meanwhile, 370 consumers whose purchasing willingness and behavior were contrary, accounting for 33.9% of the surveyed samples.

(1) *Purchasing willingness and behavior bias of the consumers with different gender*

The deviation of female consumers’ purchasing willingness and behavior was greater than that of male consumers in the surveyed farmers. There were 210 male consumers and 240 female consumers with consistent purchasing willingness and behavior. The purchasing willingness and behavior of 140 male consumers and 130 female consumers were negatively consistent. There were 200 male consumers and 170 female consumers whose purchasing willingness and behavior were contrary (Table 3).

**Table 3:** Cross analysis of gender and dependent variables.

		Behavior and willingness bias			Statistical
		Positive consistency	Disobey	Negative consistency	
Gender	Man	210	200	130	540
	Woman	240	170	140	550
Statistical		450	370	270	1090

(2) *Purchasing willingness and behavior bias of the consumers with different occupation*

The deviation of consumers’ purchasing willingness and behavior with different occupations were significantly different.

There were 150 students, 110 farming, 70 migrant workers, 80 freelance workers, and 40 other occupations who had consistent purchase willingness and behavior; There are 10 students, 70 farmers, 100 migrant workers, 70 freelancers, and 20 other professional consumers whose purchase intention and behavior are negatively consistent; There are 160 students, 70 farmers, 60 workers, 70 freelancers and 10 other professional consumers whose purchase intention and behavior are contrary to each other. Among all consumers participating in the survey, the number of students was 320, accounting for 29. 36%. The number of farmers was 250, accounting for 22.94%. Migrant workers accounted for 21.10%; There were 220 freelancers, accounting for 20.18%. There were 70 people in other occupations, accounting for 6.42%. It can be seen from the figure that among all the consumers surveyed, the number of students was the largest. Cross analysis of occupations and dependent variables were shown in Table 4. (3) *Purchasing willingness and behavior bias of the consumers with different age*.

**Table 4:** Cross analysis of occupations and dependent variables.

		Behavior and willingness bias			Statistical
		positive agreement	disobey	negative agreement	
Occupation	Students	150	160	10	320
	Farming	110	70	70	250
	Migrant workers	70	60	100	230
	freelance worker	80	70	70	220
	other	40	10	20	70
Statistical		450	370	270	1090

Among all the consumers surveyed, the consumers who were willing to and have bought, the consumers between the ages of 20 and 35 were the most, and those under the age of 20 were the least (Table 5).

**Table 5:** Cross analysis of age-dependent variables.

		Behavior and willingness bias			Statistical
		positive agreement	disobey	negative agreement	
Age	Under 20 years	10	10	0	20
	20 to 35 years old	220	210	80	510
	35 to 40years old	90	70	90	250
	40 to 45 years old	90	60	70	220
	45 years of age or older	40	20	30	90
Statistical		450	370	270	1090

(4) *Purchasing willingness and behavior bias of the consumers with different education*

Among all the consumers who participated in the survey, the most consumers had a college degree or a bachelor’s degree, and the least consumers had a graduate degree or above (Table 6). (5) *Purchasing willingness and behavior bias of the consumers with different monthly disposable income*.

**Table 6:** Cross analysis of education level and dependent variables.

		Behavior and willingness bias			Statistical
		positive agreement	disobey	negative agreement	
Education	Senior high school and below	100	40	20	160
	Junior college or undergraduate	270	290	210	770
	Postgraduate and above	80	40	40	160
Statistical		450	370	270	1090

Among all the consumers surveyed, those with a monthly income of 2,000 RMB or less had the most, while those with a monthly disposable income of 6,000 RMB or more had the least (Table 7).

**Table 7:** Cross analysis of disposable income and dependent variables.

		Behavior and willingness bias			Statistical
		positive agreement	disobey	negative agreement	
Monthly disposable income of consumers	2000 RMB of the following	180	140	20	340
	2000–4000 RMB	110	100	80	290
	4000–6000 RMB	100	60	130	290
	More than 6000RMB	60	70	40	170
Statistical		450	370	270	1090

### Regression analysis

The logistic regression model was used to analyze the impact of different factors on the consumers’ willingness and behavioral bias to purchase green agri-food products online (Table 8). Hosmer and Leme show test showed that the significance was 0.305, which indicated that the model could reliably reflect the influencing degrees of the factors on consumers’ willingness and behavioral bias for purchasing green agri-food products online. Results showed that gender, age, monthly disposable income of consumers, knowledge of online shopping for agricultural products, frequency of online purchasing, and consumers’ attention to price had a significant impact on the consumers’ willingness and behavioral bias.

**Table 8:** Model regression results.

	B	S. E.	Wald	df	Significance	Exp (B)	95%EXP (B)Confidence Interval	
							Lower bound	Upper limit
Gender **	0. 66	0. 55	1. 45	1. 00	0. 01	5. 03	0. 66	5. 62
Age**	0. 27	0. 29	0. 84	1. 00	0. 02	2. 02	0. 74	2. 33
Income**	0. 32	0. 32	1. 02	1. 00	0. 02	1. 66	0. 39	1. 35
Degree of understanding*	0. 68	0. 38	3. 29	1. 00	0. 07	1. 98	0. 95	4. 12
Online purchasing frequency***	-0. 27	0. 27	0. 94	1. 00	0. 00	1. 11	0. 45	1. 31
Price *	-0. 21	0. 21	1. 01	1. 00	0. 06	1. 12	0. 53	1. 23
constant	-0. 68	2. 19	0. 10	1. 00	0. 75	0. 50		

Note

*** indicates a significance level of 1%

** indicates a significance level of 5%

* indicates a significance level of 10%.

(1) The influence of individual characteristics of consumers. Consumers’ gender, age, and monthly disposable income positively affected their willingness and behavioral bias. The willingness and behavior of male consumers to purchase green agri-food products was more consistent than that of female consumers. Male consumers pay more attention to efficiency during the shopping, while female consumers would consider more factors during the shopping. Age also has a significant impact on consumers’ behavior and willingness to purchase agri-food products online. The younger the consumers were, the more likely they were to accept new things, and the more likely they were to accept the form of online agri-food products. Monthly disposable income also has a significant impact on consumers’ online purchasing behavior, the higher the income, the higher the frequency of online purchasing.

(2) The influence of consumer cognition degree. Degree of understanding of online consumers in the 10% of significant level affects the consumers’ online purchasing the consistency of desire and behavior of agri-food products, agri-food product online there is a certain amount of unpredictability. Therefore, consumers’ purchase behavior and willingness deviate due to the uncertainty when they choose online agricultural products. Consumers have a higher understanding of purchasing behavior, and their behaviors deviate from their wishes.

(3) The impact of consumers’ acceptance of online purchasing. Online purchasing frequency under the 1% significant level of negative affect consumers’ online purchasing willingness and behavior of agri-food products, the consistency that online purchasing frequency, the more the easier behavior willingness. Taking Beijing as an example, Ma studied the impact of consumers’ cognition of safe agri-food products and other factors on payment factors and purchasing behaviors [26]. The possible reason is that the more times consumers online, the more problems they will have and the more online purchasing experience they will have. In the case of poor purchasing experience, when consumers choose to buy agricultural products online again, they will pay attention to it, thus expanding the purchase intention and behavior bias.

(4) The impact of consumers’ preference for online purchasing of agri-food products. The price of agricultural products negatively affects the consistency of consumers’ willingness and behavior to purchase agricultural products online at a significant level of 10%, indicating that the more importance consumers attach to the price of agricultural products, the lower the consistency of their purchase willingness and behavior, and the price of green agricultural products purchased online tends to be higher than that of ordinary agricultural products due to the influence of production and logistics costs. The high price of agricultural products will reduce consumers’ purchase volume.

## Discussion

### General discussion

This paper analyzed the deviation of consumers and their subgroups in their willingness and behavior to purchase green agri-food products online, we can see that:

Firstly, the deviation of consumers’ willingness and behavior to purchase green agri-food products online was still relatively large. This is consistent with existing studies [16]. There were significant differences in the deviation of consumers’ behaviors and willingness to purchase green agri-food products online among different consumer groups.

Secondly, in terms of cognition degree of online purchasing, consumers’ understanding degree of agri-food products is generally low, and the understanding degree has a significant negative impact on the deviation of consumers’ willingness and behavior. When consumers do not often purchase green agri-food products online or have never bought agri-food products online, their understanding of online agri-food products will be very low. The less they know about agri-food products, the less they will choose online purchasing. Only when online purchasing has a full understanding of the online purchasing of agri-food product can it increase its ability to deal with purchasing risks. Naturally consumers will be more willing to purchase green agri-food products online, and the deviation of their behaviors and willingness will be smaller. This also reflects, to some extent, the positive effect of willingness on behavior [5].

Thirdly, the frequency and price of online shopping have a negative impact on the consistency of consumers’ willingness and behavior, in which the higher the frequency of online shopping, the lower the consistency of online shopping willingness and behavior, which fully illustrates the problem of incomplete regulations of online sales of agricultural products in China [11], in addition, the price of goods has a greater impact on the consistency of consumers’ online shopping behavior and willingness. Consumers pay more attention to the prices of agricultural products in online shopping, and the products with affordable price are more likely to be favored by consumers, online shopping for agricultural products with good quality and low prices can increase consumers’ motivation. This is a reflection of consumers’ reduced perceived risk and increased perceived value, which is conducive to increasing the willingness to purchase online [27].

### Suggestions

Although the e-commerce market of agri-food products has not started for a long time, the rapid development of e-commerce of agri-food products breaks the geographical restrictions of the traditional market, shortens the time for agri-food products from the field to the dining table, and consumers can enjoy high-quality agri-food products without leaving home. What the e-commerce of agri-food products has is that the e-commerce of agri-food products has attracted the attention of many agri-food products sellers as soon as it came into being, But the competition is fierce. However, fundamentally speaking, the most important thing to do well in an e-commerce platform for agri-food products is to capture consumers. Based on the analysis of factors influencing the purchase behavior and willingness of agri-food products, the following enlightenments can be obtained.

First of all, because of the large deviation of consumers’ willingness and behavior, if they are inconsistent, online sellers of agri-food products can not completely rely on consumers’ purchase willingness when formulating sales strategies. The seller should actively expand the market instead of completely dependent on online sales, and also should be properly set up some offline sales sites.

Secondly, according to the characteristics of different consumer groups and the obvious differences between groups, we can analyze the personal characteristics of consumers to make a higher level of market segmentation for consumer groups, and divide the original market into more sub-markets to make the marketing and publicity for consumers more targeted, aiming to increase the consumption stickiness of agri-food products, and expand the market share horizontally and vertically.

Thirdly, given the problem that consumers’ brand awareness of agricultural products is generally low, network operators should pay attention to the brand promotion of agricultural products, improve consumers’ awareness of agricultural products through network new media and other channels. Agricultural products and agricultural departments should improve the credibility and awareness of agricultural products through holding meetings, exhibitions, marketing, and other activities, to improve the competitiveness of agricultural products Stimulate consumers’ online demand for agricultural products, make consumers feel the convenience and benefits of online purchase of agricultural products, and speed up the construction of agricultural products.

Fourthly, in response to the problem of the poor experience of consumer online shopping for agricultural products fee for the low trust of online shopping for agricultural products, relevant departments should first actively improve the quality certification standards and quality testing mechanism for agri-food products. It is necessary to constantly improve relevant laws and regulations on the quality and sailing procedures of agri-food products sailed online, establish unified standards on the quality and safety of agri-food products, and strengthen the supervision and management of agri-food product safety certification organizations, to improve the supply quality of agri-food product in China. Only by providing high-quality and food safety standards, e-commerce agricultural products can stimulate consumers’ purchasing intention and form a good reputation on the Internet [28].

Fifthly, it has an important influence and deviation on the price of agricultural products and consumers’ online purchase of agricultural products. Agricultural institutions can provide production subsidies for high-standard agricultural products. At the same time, relevant government departments, chambers of e-commerce, cooperatives, and other institutions can provide some technical guidance services for farmers, help farmers reduce production costs, provide promotional services for farmers, and reduce the cost of farmers’ sales, so that high-quality agricultural products can be fixed in the sales, the price is more reasonable, to attract consumers to buy.

### Limitations and research prospects

In this study, the theoretical framework was constructed by using more existing indicators, and more micro indicators were selected, while the macro social environment indicators were neglected. For example, the impact of the new pneumonia epidemic on consumers’ online purchase of green agricultural products should be set to improve the theoretical framework. In addition, the empirical research results of this paper are based on the survey respondents, and it is necessary to further test whether the results can remain relatively consistent after the research sample changes, which also provides ideas for future research.
